# Overweight as a Prognostic Factor for Triple-Negative Breast Cancers in Chinese Women

**DOI:** 10.1371/journal.pone.0129741

**Published:** 2015-06-24

**Authors:** Shuang Hao, Yin Liu, Ke-Da Yu, Sheng Chen, Wen-Tao Yang, Zhi-Min Shao

**Affiliations:** 1 Department of Breast Surgery, Fudan University Shanghai Cancer Center, Shanghai, China; 2 Department of Oncology, Shanghai Medical College, Fudan University, Shanghai, China; 3 Department of Pathology, Fudan University Shanghai Cancer Center, Shanghai, China; 4 Institutes of Biomedical Science, Fudan University, Shanghai, China; 5 Key Laboratory of Breast Cancer in Shanghai, Department of Breast Surgery, Fudan University Shanghai Cancer Center, Shanghai, China; University of South Alabama, UNITED STATES

## Abstract

**Purpose:**

Obesity is associated with poorer outcomes in patients with hormone receptor-positive breast cancers, but this association is not well established for women with triple-negative breast cancers (TNBC). Here, we investigated the prognostic effects of body mass index (BMI) on clinical outcomes in patients with TNBC.

**Methods:**

We identified 1106 patients with TNBC who met the inclusion criteria and were treated between January 2002 and June 2012. Clinical and biological features were collected to evaluate the relation between BMI and breast cancer-specific survival (BCSS) and overall survival (OS) after controlling for other clinically significant variables.

**Results:**

Of 1106 patients, 656 (59.3%) were normal weight (BMI ≤24) and 450 patients (40.7%) were overweight(BMI>24). Median follow-up time was 44.8 months. Breast cancer specific death was observed in 140 patients. After adjusting for clinicopathologic risk factors, overweight was associated with OS (hazard ratio [HR]: 1.46, 95% confidence interval [CI]: 1.04-2.06, *P* =0.028) but not BCSS (HR: 1.34, 95% CI: 0.90–2.01, *P* =0.15)in all the patients with TNBC. When stratified with menopausal status, overweight was associated with BCSS and OS (HR: 2.27, 95% CI: 1.11-4.63, *P* = 0.024 and HR: 2.16, 95% CI: 1.21-3.87, *P* = 0.010, respectively) in premenopausal women. BMI was not associated with BCSS or OS in postmenopausal women.

**Conclusions:**

Overweight is an independent prognostic factor of OS in all women with TNBC, and menopause status may be a mitigating factor. Among premenopausal women, overweight women are at a greater risk of poor prognosis than normal weight women. If validated, these findings should be considered in developing preventive programs.

## Introduction

Gene expression profiles have reshaped our understanding of breast cancer by defining and characterizing four main subtypes: human epidermal growth factor receptor-2 (HER2)-enriched, basal-like, luminal A, and luminal B[1]. Basal-like breast cancer has a unique clinical-pathological presentation, prognosis, and response to therapy, with poorer prognosis than luminal tumors, but can be difficult to identify in clinic. About three quarters of triple-negative breast cancers (TNBCs)—i.e., estrogen receptor (ER)^-^, progesterone receptor (PR)^-^ and not overexpressing HER2[2]—express basal markers, so the triple-negative type is frequently taken as a surrogate marker of basal-like breast cancer.

For 30 years, accumulating evidence suggests that obese women have poorer prognoses than lean ones after breast cancer treatment[3–5]. In an observational prospective study of about 350,000 US women, higher BMI was very significantly associated with increasing risk of dying from breast cancer[6]. However, very few studies, all with small samples, have investigated the influence of body mass index (BMI) in TNBC outcomes[7]. Consequently, we examined the impact of overweight on breast cancer-specific survival (BCSS) and overall survival (OS)in Chinese patients treated for TNBC between January 2002 and June 2012.

## Patients and Methods

### Study population

From the database of Fudan University Shanghai Cancer Center, we identified 1,106 women with TNBC with complete follow-up data who received treatment for early-stage breast cancer. According to the inclusion criteria, all cases were confirmed as females with TNBC but no distant metastasis at the initial diagnosis. All patients received a complete physical examination, bilateral mammography, chest radioscopy, ECG and ultrasonography of the breast, axillary fossa, abdomen and pelvis. All patients at risk for relapse received adjuvant chemotherapy, using different regimens according to the standards used at the time of surgery, followed by radiotherapy (if required). Exclusion criteria included ER or PR positivity, HER2 overexpression or amplification, unknown date of surgery, absence of date of last follow-up, additional malignancy and male sex. Information was available for all patients’ age and menopausal status at diagnosis, tumor size, number of lymph nodes removed, number of positive lymph nodes, histological type, histological grade and treatment regimen. Data on height and weight at diagnosis to compute BMI were also available for these patients. We identified 24 kg/m^2^as the cut-off point according to the Working Group on Obesity in China guideline[8].

Information on date and cause of death came from linking to Center of Disease Control records, using the unique personal identification number issued to every Chinese citizen. Causes of death were obtained from death certificates and were classified as either death as a result of breast cancer or death from other causes. In this study, we identified BCSS and OS as the time-to-event end points. BCSS was defined as time from surgery to death as a result of breast cancer. OS was calculated from the date of surgery to the date of death irrespective of any cause or last follow-up.

The study was conducted according to the principles expressed in the Declaration of Helsinki and approved by the institutional review board of Fudan University Shanghai Cancer Center. All the patients enrolled in this study signed the informed consent voluntarily.

### Histopathology and immunohistochemistry

ER, PR and HER2 status were determined on representative paraffin sections of each tumor, using immunohistochemical (IHC) staining, performed after the patient underwent surgery. ER and PR antibodies were purchased from Dako (clones ER 1D5 1:35 and PR 636 1:50) and were evaluated by an avidin–biotin–peroxidase complex (ABC) assay as described by Shimada et al[9]. ER and PR were considered positive in breast cancer cells if the positive nuclei number was ≥10%. After July 2010, tumors in which ≥ 1% of tumor cells staining for ER or PgR of any intensity were considered positive[10]. Cytoplasmic staining was ignored[11]. Overexpression of HER2 protein was evaluated using a monoclonal antibody (Dako, Clone PN2A 1:400) and a peroxidase-antiperoxidase (PAP) technique. Positive HER2 was defined as a complete membrane staining in >10% of tumor cells[12], using a qualitative HercepTest scale of 0–3+, in which scores 0–1 were negative, and 3 was positive[13]. Fluorescence *in situ* hybridization tests were used when the IHC results were ambiguous (i.e., 2+), or for patients who could not be defined as HER2^-^. The pathological and IHC outcomes were diagnosed under an Olympus light microscope with ×10 and ×40 magnifications by two independent pathologists in the Department of Pathology, Fudan University Shanghai Cancer Center.

### Statistical analysis

Associations between BMI and other characteristics were analyzed using the chi-square test. For the assessment of the influence of BMI on survival outcome, we adjusted for age at diagnosis, menopausal status, tumor size, nodal status, grade and systemic adjuvant therapy by multivariate Cox proportional hazards models. The assumption of Cox’s proportional hazards model was assessed by the analysis of Schoenfeld residuals [14]. All *P* values were two sided. Statistical analyses were performed using SPSS Statistics 19.0 (IBM SPSS Statistics 19). *P*< 0.05 was considered significant.

## Results

### Characteristics of patients

Of the 1106 patients, 656 (59.3%) were normal weight (BMI≤24), and 450 patients (40.7%) were over weight(BMI>24). Median follow-up time was 44.8 months. Breast cancer specific death was observed in 140 patients. The normal weight group’s median age was 50 years (range: 23–85 years). The overweight group’s median age was 53 years (range: 30–86 years). The overweight group had a significantly higher proportion of larger tumors (>2cm; *P<*0.001), included more postmenopausal women (*P<*0.001), and less patients received chemotherapy than in the normal weight group (*P* = 0.009; Table 1).

**Table 1:** Baseline characteristics of patients.

Characteristics^a^	Total	BMI≤24	BMI>24	*P*
	*N* (%)	*N* (%)	*N* (%)	
Tumor size (cm)				
≤2.0	456 (41.9%)	294 (45.9%)	162 (36.2%)	<0.001
>2.0	632 (58.1%)	346(54.1%)	286(63.8%)	
Tumor grade				
I–II	462 (43.4%)	280 (44.9%)	182 (41.7%)	0.256
III	602 (56.6%)	344 (55.1%)	258 (58.3%)	
LN status				
-	652 (59.0%)	380 (57.9%)	272 (60.4%)	0.403
+	454 (41.0%)	276 (42.1%)	178 (39.6%)	
Menopausal status				
Pre-	568 (51.4%)	370 (56.4%)	198 (44.0%)	<0.001
Post-	538 (48.6%)	286 (43.6%)	252 (56.0%)	
Chemotherapy				
-	64 (5.8%)	28 (4.3%)	36 (8.0%)	0.009
+	1042 (94.2%)	628 (95.7%)	414 (92.0%)	

BMI: body mass index; LN: lymph node.

^a^The numbers of tumor size, tumor grade and LN status were less than the total number of subjects because some clinical data were missing.

BMI was not associated with tumor grade and lymph node status(Table 1).

### Overweight and survival

**Fig 1:**
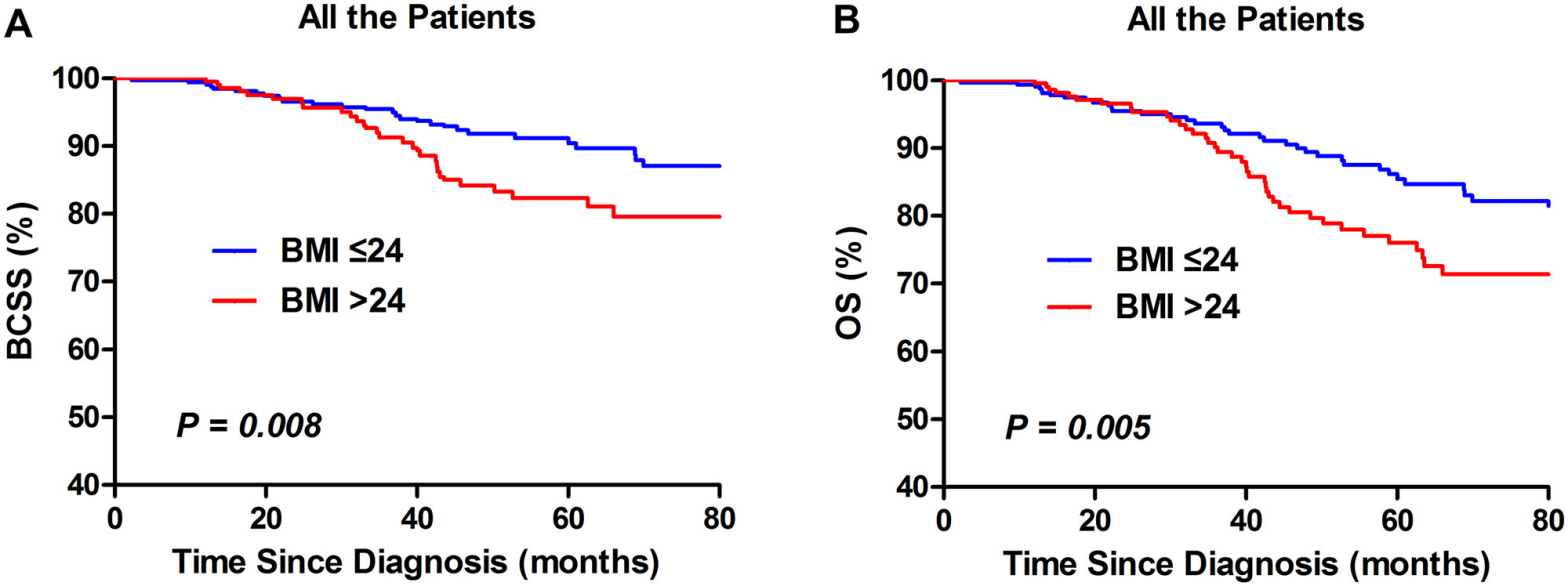
Kaplan–Meier curves of BCSS (A) and OS (B) by BMI groups in all TNBC patients.

**Fig 2:**
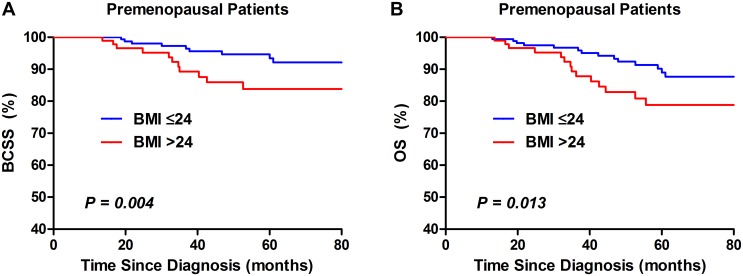
Kaplan–Meier curves of BCSS (A) and OS (B) by BMI groups in premenopausal TNBC patients.

**Fig 3:**
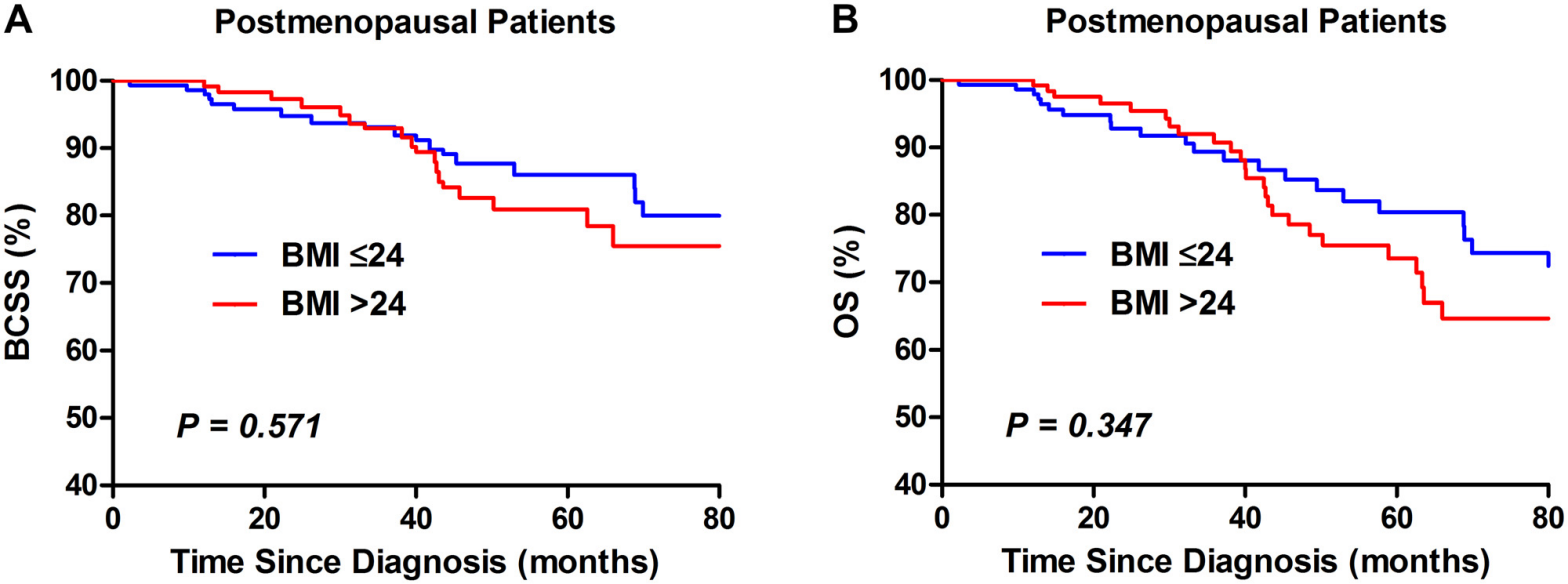
Kaplan–Meier curves of BCSS (A) and OS (B) by BMI groups in postmenopausal TNBC patients.

Univariate analysis using the Kaplan-Meier method indicated that overweight was significantly (*P* = 0.008) associated with poorer BCSS and OS in TNBC patients (Fig 1A and 1B). After stratified by menopausal status, overweight indicated poorer BCSS (*P* = 0.004; Fig 2A) and OS (*P* = 0.013; Fig 2B) in premenopausal women, but not postmenopausal ones (*P* = 0.571and*P* = 0.347; Fig 3A and 3B).

In multivariable analysis, overweight was significantly with OS(hazard ratio [HR]:1.46, 95% confidence interval [CI]: 1.04–2.06, *P* = 0.028) but not BCSS (HR: 1.34, 95% CI: 0.90–2.01, *P* = 0.150)after adjustment for clinicopathologic risk factors(Table 2). In stratification analysis, overweight was an independent prognostic factor for BCSS (HR:2.27, 95% CI: 1.11–4.63, *P* = 0.024; Table 3) and OS (HR: 2.16, 95% CI: 1.21–3.87, *P* = 0.010; Table 4). Among postmenopausal women, BMI did not predict for BCSS (HR: 0.96, 95% CI: 0.58–1.58, *P* = 0.861;Table 3) or OS (HR: 1.07, 95% CI: 0.70–1.64, *P* = 0.754;Table 4). By using the Schoenfeld residuals, there was no evidence that the effect of BMI on survival outcome changed with date of diagnosis (*P* = 0.928) in the test of proportional hazards assumption.

**Table 2:** Multivariate survival analysis in TNBC patients.

Menopausal status	BCSS			OS		
	*P* ^a^	HR^a^	95% CI	*P* ^a^	HR^a^	95% CI
Total						
Tumor size	<0.001			<0.001		
T1		1.00			1.00	
T2		1.03	0.66–1.63		1.05	0.72–1.52
T3		7.01	3.94–12.46		5.50	3.29–9.20
Nodal status	<0.001	5.54	3.36–9.14	<0.001	3.53	2.44–5.11
BMI	0.150	1.34	0.90–2.01	0.028	1.46	1.04–2.06
Chemothearpy	0.028	0.50	0.27–0.93	NS		
Grade	NS			NS		
Premenopausal						
Tumor size	<0.001			<0.001		
T1		1.00			1.00	
T2		1.45	0.63–3.34		1.11	0.59–2.07
T3		7.48	2.81–19.92		4.78	2.11–10.80
Nodal status	<0.001	12.35	4.30–35.42	<0.001	5.77	2.99–11.14
BMI	0.024	2.27	1.11–4.63	0.010	2.16	1.21–3.87
Chemotherapy	NS			NS		
Grade	0.039	0.49	0.25–0.96	NS		
Postmenopausal						
Tumor size	<0.001			<0.001		
T1		1.00			1.00	
T2		0.85	0.49–1.49		1.02	0.64–1.62
T3		7.29	3.37–15.78		7.87	3.89–15.90
Nodal status	<0.001	3.95	2.19–7.15	<0.001	2.59	1.63–4.11
BMI	0.861	0.96	0.58–1.58	0.754	1.07	0.70–1.64
Chemotherapy	NS			NS		
Grade	NS			NS		

BMI: body mass index; CI: confidence interval; HR: hazard ratio.

^a^Adjusted for age at diagnosis, menopausal status, tumor size, nodal status, grade and systemic adjuvant therapy

**Table 3:** Univariate and multivariate BCSS analysis of BMI in TNBC patients according to menopausal status.

Menopausal status/BMI	Patients *N*	Events *N*	Univariate *P*	Multivariate *P* ^a^	HR^a^	95% CI
Total	1106	106	0.008	0.150	1.34	0.90–2.01
BMI≤24(normal weight)	656	50				
BMI>24(overweight)	450	56				
Premenopausal patients						
Total	568	40	0.004	0.024	2.27	1.11–4.63
BMI≤24(normal weight)	370	18				
BMI>24(overweight)	198	22				
Postmenopausal patients						
Total	538	66	0.571	0.861	0.96	0.58–1.58
BMI≤24(normal weight)	286	32				
BMI>24(overweight)	252	34				

BCSS: breast cancer-specific survival; BMI: body mass index; CI: confidence interval; HR: hazard ratio. NS: no significance

^a^Adjusted for age at diagnosis, menopausal status, tumor size, nodal status, grade and systemic adjuvant therapy

**Table 4:** Univariate and multivariate OS survival analysis of BMI in TNBC patients according to menopausal status.

Menopausal status/BMI	Patients *N*	Events *N*	Univariate *P*	Multivariate *P*	HR	95% CI
Total	1106	154	0.005	0.028	1.46	1.04–2.06
BMI≤24(normal weight)	656	76				
BMI>24(overweight)	450	78				
Premenopausal patients						
Total	568	58	0.013	0.010	2.16	1.21–3.87
BMI≤24 (normal weight)	370	30				
BMI>24(overweight)	198	28				
Postmenopausal patients						
Total	538	96	0.347	0.754	1.07	0.70–1.64
BMI≤24 (normal weight)	286	46				
BMI>24(overweight)	252	50				

BCSS: breast cancer-specific survival; BMI: body mass index; CI: confidence interval; HR: hazard ratio. NS: no significance

^a^Adjusted for age at diagnosis, menopausal status, tumor size, nodal status, grade and systemic adjuvant therapy

## Discussion

Patients with TNBC tend to have worse clinical outcomes partly as a result of lacking a therapeutic target. Consequently, establishing a relation between modifiable factors that may portend an adverse outcome potentially may be beneficial to these patients[15]. This study confirms reports that overweight patients often have larger tumors at diagnosis[6, 16], and shows BMI >24 to independently predict BCSS in premenopausal TNBC patients, but not in postmenopausal ones after adjusting for these known prognostic factors. Overweight also had a negative influence on OS, especially in premenopausal patients.

Although many studies with sufficiently large cohorts have found associations between obesity and patient survival[17–20], very few studies have focused on BMI’s prognostic role in TNBC, the results of which vary considerably[7, 15, 21–24]. The results of our study are consistent with several reports in the literature, which suggest that increasing BMI was associated with inferior survival in TNBC. In a single center study of 818 patients with TNBC, OB was associated with worse DFS and OS for premenopausal patients with TNBC at a median follow-up of 29 months[22]. Similar findings were observed in a pooled analysis of 8,872 patients, which had a median follow-up of 42.7 months. Mean DFS and OS were shorter in obese and very obese compared with normal weight patients in TNBC[25]. Pathological complete response rate was higher in normal weight patients compared with overweight groups. The impact of BMI on breast cancer outcome has also been investigated by Pajares et al. in 5,683 operable breast cancer patients enrolled in four randomized clinical trials. In this pooled analysis, severely obese patients treated with anthracyclines and taxanes present a worse prognosis regarding DFS, BCSS and OS than patients with BMI < 25across subtypes including TNBC[26].

Tait et al. [22] suggested that BMI did not affect survival for patients with TNBC. Ademuyiwa et al. found no significant relationship between obesity and RFS or OS in patients with TNBC after controlling for clinically significant factors[15], possibly because TNBC patients tend to receive cytotoxic chemotherapy which may neutralize potential detrimental effects of a higher BMI. Both of these studies included a smaller sample size compared to our study. To date, two largest pooled analyses supported a negative effect of BMI on survival outcomes in patients with TNBC[25, 26]. Our data provided critical additional evidence for this finding.

High BMI might influence TNBC prognosis through various mechanisms, such as obesity-associated comorbidities that interfere with optimal treatment, the effect of higher cholesterol level on tumor metastasis, or other mechanisms. High BMI might cause higher cholesterol levels and even fatty liver disease, which would affect implementation or dosing of chemotherapy. Nelson et al. demonstrated that liver X receptor activation by 27-hydroxycholesterol (27HC, a primary cholesterol metabolite) increases tumor metastasis and that these activities occur independently of ER[27]. Consequently, obesity or OW, which cause higher 27HC levels, could indicate poorer prognosis. Leptin, the adipose tissue cytokine, increases in obesity and promotes survival of cancer stem cells in vivo, consequently promoting breast cancer[28]. Moreover, Howe et al found out that saturated fatty acids, released as a consequence of obesity-associated lipolysis, induce macrophage activation via Toll-like receptor-4, thereby stimulating NF-κB signaling. This, in turn, activates transcription of pro-inflammatory factors including COX-2, IL-6, IL-1β, and TNFα[29], which promote tumor growth and progression through various mechanisms.

In the multivariate analysis, higher BMI was associated with OS rather than BCSS in the whole study population. Previous studies turned out that obesity is probably the most powerful modifiable risk factor for the incidence and prognosis of breast cancer. Obesity is well known to be a potent promoter of comorbid conditions, as cardiovascular risk factors including glucose and lipid disorders and elevated blood pressure for instance, which may reduce the patients’ overall survival more significantly. When the association was evaluated based on women’s menopausal status, data were significant only among pre-menopausal individuals, suggesting that distinct molecular mechanisms are involved in the onset and progression of TNBC during the women’s reproductive life. Why BMI is an independent predictor in premenopausal TNBC patients but not in postmenopausal ones is unclear. TNBC is a molecularly and clinically heterogeneous disease, which may be genomically subdivided into 3 distinct tumors: those with HER2 gene amplification; basal-like tumors; and tumors without ER, PR, HER2, or basal-like features[30]. TNBC diagnosed in younger patients might differ from than in older ones.

To our knowledge, this is the first study to examine the relationships between obesity and TNBC survival outcomes in a relatively large Chinese patient cohort. There were some potential limitations in our study. Firstly, due to the relatively proportion of underweight and obese patients, classification was measured in a binary scale. This classification did not allow to clearly distinguish between women who were obese and those who were overweight. Secondly, the current study population is composed of 1106 early-stage TNBCs received surgical treatment. Meanwhile, neoadjuvant treatment is an important treatment option in TNBC patients, assessing the prognostic and predictive impact of BMI in the neoadjuvant setting is essential and warranted. Moreover, our analysis was limited to homogeneous Chinese population, the generalize ability to other ethnic groups is uncertain.

In conclusion, according to this analysis, overweight women with TNBC are at a greater risk of poor prognosis than normal weight women in premenopausal population. If validated, these findings should be taken into consideration for the development of preventive programs in TNBCs.

## Supporting Information

S1 Data Data set underlying the findings. (XLSX)

S1 Table Summary of the similar papers and ours. (DOCX)
