# Supplementary material for: Overweight as a Prognostic Factor for Triple-Negative Breast Cancers in Chinese Women
Source: PLoS One. 2015 Jun 24;10(6):e0129741. doi: 10.1371/journal.pone.0129741 (PMC4479880; doi:10.1371/journal.pone.0129741)
Supplement: S1 Table — (DOCX) [file pone.0129741.s002.docx]

| **Author** | **Objective** | **Study design** | **Race** | **TNBC No.** | **Subtypes** | **Endpoint** | **Follow-up (months)** | **Primary treatment** | **BMI classification** | **Overweight (%)** | **Conclusion** |
| --- | --- | --- | --- | --- | --- | --- | --- | --- | --- | --- | --- |
| Tait | evaluate the relationship between obesity and diabetes with TNBC survival outcomes | retrospective | African American and Caucasian | 488 | TNBC | DFS, OS | 40.1 | Surgical treatment | <25, 25.0-29.99, 30-34.99, ≥35 | 75.9 | Obesity and diabetes did not significantly affect survival for patients with TNBC |
| Chen | evaluate whether obesity is a risk factor for different breast cancer subtypes | retrospective | Chinese | 477 | TNBC, luminal A, luminal B, Her-2+ | Occurrence | NA | Surgical treatment | <18.5, 18.5-25, 25-30, >30 | 73.3 | the overweight and obesity groups are associated with increased TNBC risk |
| [Turkoz](http://www.ncbi.nlm.nih.gov/pubmed/?term=Turkoz%20FP%5BAuthor%5D&cauthor=true&cauthor_uid=23818343) | evaluate the impact of obesity on tumor features, hormonal status, recurrence and mortality in premenopausal breast cancer patients | retrospective | Caucasian | 122 | TNBC, luminal A, luminal B, Her-2+ | DFS, OS | 29.0 | NA | 18.5-25, 25-30, >30 | NA | Obesity is associated with ER and PR negative tumors and poor OS in premenopausal women with breast cancer |
| Fontanella | evaluate the impact of obesity on neoadjuvant treatment outcome | pooled analysis | Caucasian | 3282 | TNBC, luminal-like, HER2/luminal, HER2-like | DFS, OS, pCR | 42.7 | Neoadjuvant | <18.5, 18.5-24.99, 25-29.99, 30-39.99, ≥40 | 50.9 | Mean DFS and OS were shorter in obese and very obese compared with normal weight patients which was consistent in luminal-like and TNBC.Higher BMI was associated with lower pCR and a detrimental impact on survival. |
| Pierobon | evaluate the association between obesity, menopause status and TNBC risk | meta analysis | NA | 3845 | TNBC, non-TNBC | Occurrence | NA | NA | <30,≥30 | 26.7 | High BMI represents a risk factor for non-TNBC especially in post-menopausal women. In the pre-menopausal group, a significant association between BMI and TNBCs was observed. |
| Jain | evaluate the impact of obesity on breast cancer incidence, recurrence and mortality. | review | NA | NA | NA | NA | NA | NA | NA | NA | Obese women had a 33 % increased risk of both breast cancer-specific and overall mortality |
| Hao | evaluate the impact of obesity on breast cancer-specific survival in Chinese patients treated for TNBC | retrospective | Chinese | 1106 TNBCs | TNBC | BCSS | 44.8 | Surgical treatment | ≤24, >24 | 40.7 | Overweight is a prognostic factor for TNBCs in premenopausal Chinese women |

**Supporting Information Table S1** summary of the reviewer’s mentioned papers and ours
